# Marine Parasites as Bioindicators for Toxins and Their Potential Role as Environmental Sinks for Marine Fauna

**DOI:** 10.1155/japr/5538084

**Published:** 2026-07-05

**Authors:** Anshika Yadav, Anita Yadav, Payal Mahobiya, Sandeep K. Malhotra

**Affiliations:** ^1^ Department of Zoology, Dr. H.S. Gour Sagar Vishwavidyalaya, Sagar, Madhya Pradesh, India; ^2^ Department of Zoology, C.M.P.P.G. College (a Constituent College of the University of Allahabad), Prayagraj, Uttar Pradesh, India; ^3^ Department of Zoology, University of Allahabad, Prayagraj, Uttar Pradesh, India, allduniv.ac.in

**Keywords:** anisakidosis, bioindicator, EDX microanalysis, environmental pollution, heavy metals, metal filter, *Rotundocollarette capoori*, scanning electron micrography, zoonotic

## Abstract

**Background:**

Researches are being frequently conducted on the utility aspects of helminth parasites worldwide. The evolution is driven by the need for survival against the toxicity of biometals that emerged in zoonotic roundworms of Anisakidae.

**Aims:**

This study is aimed at investigating whether the sequestration of biometals from the environment into the body tissues of parasites serves a dual purpose: firstly, the removal of toxic metals and other biotoxins from the immediate environment of worms and, secondly, the utilization of extra available biometals, like sulfur and cysteine, in the surrounding environment to strengthen the cuticularization for the body′s defense. Additionally, it examines whether the toxins absorbed by the tissues of worms thus protect tissues of the fish host from damage caused by toxicity; the potential “environmental sink” thus came into operation.

**Methods:**

These worms exploited the alterations in chemical composition to adapt and evolve in the newer environment. An assessment of biotoxic influence generated through heavy metal toxicity was conducted during this investigation. Scanning electron microscopy and energy‐dispersive x‐ray micrographic analysis were the techniques employed. The studies were conducted in anisakid nematodes parasitizing reef‐associated fishes at Ilha “Grande” Island, 19 km from Panaji, Goa, in India.

**Results:**

The first record of *Anisakis typica* third‐stage larvae has recently appeared in Indian fish. The survival of worms of Anisakidae under the influence of a hostile environment comprising toxic biometals, like Hg, Pb, and Cd, was analyzed. The hypothesis of the metal filter being functional at the worm–iron influx interface was propounded. The typical anisakids of the family Anisakidae trigger a zoonotic mechanism that involves damage to human health.

**Conclusions:**

The superfluous iron influx within the intestinal environment apparently brought about physiological changes in the host′s body tissues as well. The excessive iron content from the tissues of nematodes first crossed the worm′s membrane barrier. Next, the iron content was stored in different body organs of roundworms to avoid the influence of toxicity on the fish host′s body. Apparently, the parasitic tissues of the nematode functionally operated as an environmental sink which could relieve body tissues of the host fish due to unwarranted duress under biometallic stress.

## 1. Introduction

Anisakiasis is the known state of pathogenesis whose causative agents are primarily the members of Anisakidae, that is, *Anisakis* and related genera. Cetaceans are usually their definitive hosts, mainly reported from marine habitats. However, rarely, the anisakids have been reported from freshwater riverine habitats in India [1]. There have been no reports on anisakid species infections in the Gangetic riverine ecosystem up until the past century, and the world literature [2–4] provides testimony to that. Recent studies [5] have discovered third‐stage larvae of *Anisakis typica* from an Indian marine fish, *Sillago sihama*. The anadromous fish, for example, *Bagarius bagarius*, the intermediate hosts for members of the family Anisakidae, are of immense economic significance, coupled with their sociocultural and ecological importance [6]. They have the potential to transport larvae and other developmental stages by traversing almost a 1000 km stretch between Hugli (in the vicinity of the Bay of Bengal) and Saraswati Ghat at Prayagraj, Uttar Pradesh. Their habit of reproductive upstream migration could thus assist in the transport of larvae and other developmental stages to Prayagraj and Fatehpur.

*B. bagarius* is not known as a migratory fish in published literature [6], but the evidence provided in this investigation of the possibility of transport of larvae of anisakids corroborated long‐distance movement of these fish for enhanced prevalence of nemic infections in this geographical area.

The Goan and Kerala coasts (neighboring states of Goa) in India are favorite spots for international tourism. The consumption of dried and uncooked fish is a most relished dish; thereby, human consumption keeps alive the chances of transmission by breaking the active salinity barrier.*Anisakis simplex* s.s. (Rudolphi), *A. typica* (Dujardin), and *Anisakis pegreffii* (Campana‐Rouget and Biocca) have been some of the most significant agents of zoonoses that contributed immeasurably to enhanced clinical cases all over the world [7–9]. A great variety of copepods are utilized by anisakid worms as intermediate hosts.

The data on stock delineation based on the response to specific water chemistry interrelationships with infective stage variations of parasitic larvae, using the roundworm *Rostellascaris spinicaudatum* in marine fish, were published [10]. The emergence of *Rotundocollarette capoori* [11] thus introduced a parasitological indicator species which provided critical inputs to the evolutionary processes of nematodes.

This major environmental disaster, such as a tsunami, triggered the disappearance of worms of camallanid genera like *Paracamallanus tridenti* [12], accompanied by a sudden stock decline of their hosts, *Lutjanus malabaricus*, which too were replaced by *Johnius dussumieri*, in which the transformed advanced version of roundworms, that is, *R. capoori*, has now emerged within the Arabian Sea. This has been one of the noticeable events. This evolution is driven by the worm′s need to survive under the influence of a hostile environment comprising Hg, Pb, Cd, etc., where they utilize toxins and other chemicals like sulfur and cysteine, for the purpose of strengthening cuticularized elements [11]. Thus, they exploit the alterations in chemical composition to adapt and evolve in the newer environment.

The toxic metals, like Hg, Pb, and Cd, that are essentially not required to accomplish completion of metabolic activities of animals, as well as those metals, like Zn, Fe, Ni, and Cu, that are required for biological metabolism, interact within the micro‐ as well as macroenvironment of a broad spectrum of helminthic worms, particularly nematodes, acanthocephalan, tapeworms, and trematodes. The worms parasitizing various organs associated with the alimentary canal and accessory glands are, by now, recognized biomonitoring tools used to detect heavy metal pollution in marine and freshwater ecosystems worldwide [13–16].

The status of a beneficial organism to these parasitic helminths in hillstream, marine, and freshwater fishes [17] has been assigned due to their potential to accumulate toxic metals, even in trace quantities. These worms are recognized as sentinel organisms that are helpful in deriving conclusions on bioremediation from an environmental perspective. There are a few studies that significantly distinguish the role of nematodes as bioindicators in the marine environment in India [10]. Among flatworms, the trematode organisms of black spot disease in hillstream fish have been distinguished as bioindicators within Indian high‐altitude zones [18]. The roundworms of Anisakidae are the common inhabitant of deep waters in meso‐ and benthopelagic fish and are specifically found to parasitize predator fishes. The current investigation envisages the application of energy‐dispersive x‐ray microanalysis (EDXMA) for the precise monitoring of the toxic metals from the tissues of worms which are retained, at times, in greater quantities than in the body organs of their respective fish hosts.

The analysis of nemic transmission pathways in view of reports of bioinvasion [10, 19] was of interest. The intriguing salinity barrier was made more inexplicable by pollutants and toxins that had to be traversed by parasitic species invading fish definitive hosts in large water bodies. The potential of metallic absorption by certain anisakid parasites in freshwater as well as marine water fish is understood to be much too enormous [11, 20] than the tissues of their hosts [11, 20]. This study would facilitate understanding of the mechanism of differential strength of absorption of biometallic ions in a variety of organs as well as body tissues during host–parasite interactions. This potential could make them beneficial to their hosts, as the latter could be protected from the toxic influence of metal alterations in the environment.

### 1.1. Anthropogenic Pollution

This pertains to the variety of pollution which occurred due to man‐made alterations in the environment. The elements would include waste products from industrial processes, vehicular emissions, etc., excluding events like volcanic eruptions that contributed to natural pollution. Greenhouse gases from fossil fuels and excess nutrients in water emanating from strategic industrialization for human development are key examples of it.

## 2. Materials and Methods

### 2.1. Site for Collection

The Jetti at Porvorim, Panaji, North Goa (15.505082°N and 73.834789°E for latitude and longitude, respectively), was the area chosen to conduct fish catches for parasitological collections. This was in the vicinity of the recently discovered coral reef–associated Ilha “Grande” Island on March 5, 2024, by Indian scientists. Its site map is given in Figure 1a.

Figure 1(a) Map of the site of investigations at Goa. (b) SEM of the head of *Rotundocollarette capoori* (dorsal view) (the EDXMA‐generated purple square represents the measured area). (c) SEM of the posterior part of the body of *Rotundocollarette capoori* (the EDXMA‐generated purple square represents the measured area) with phasmid viewed on the sublateral side of the tail. (d) SEM of the head of *Rotundocollarette capoori* (en face view) (the EDXMA‐generated purple square represents the measured area).

**Figure:**
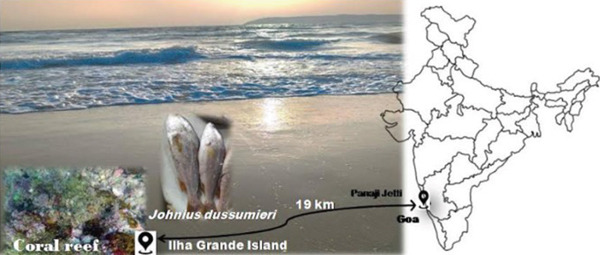
(a)

**Figure:**
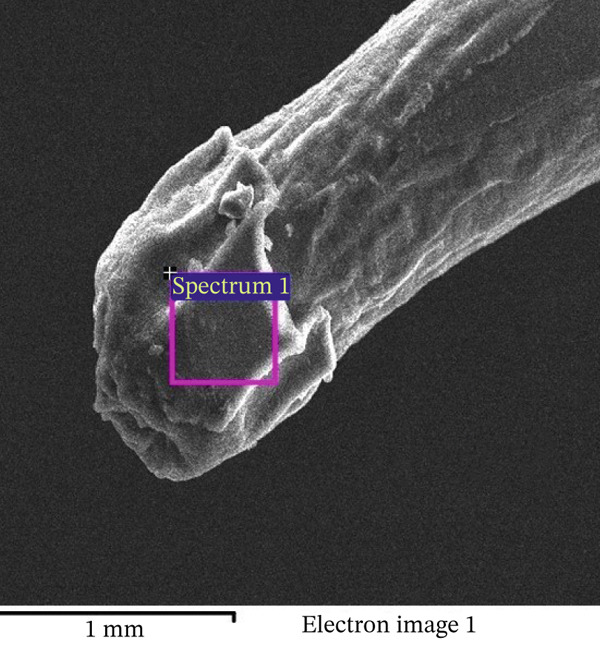
(b)

**Figure:**
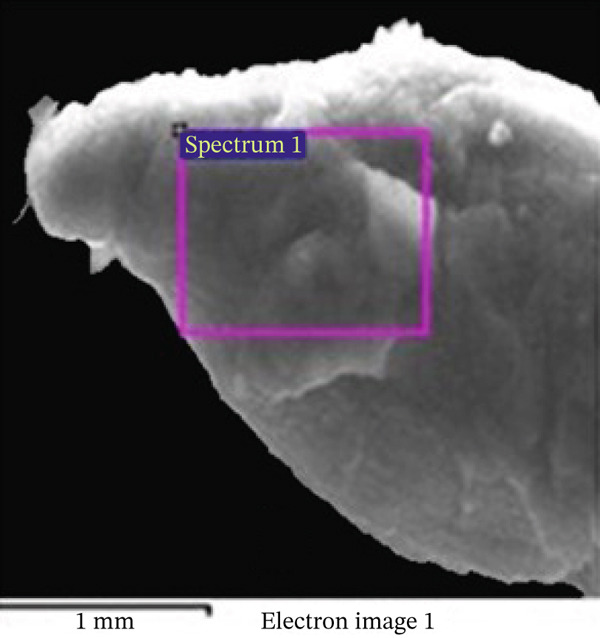
(c)

**Figure:**
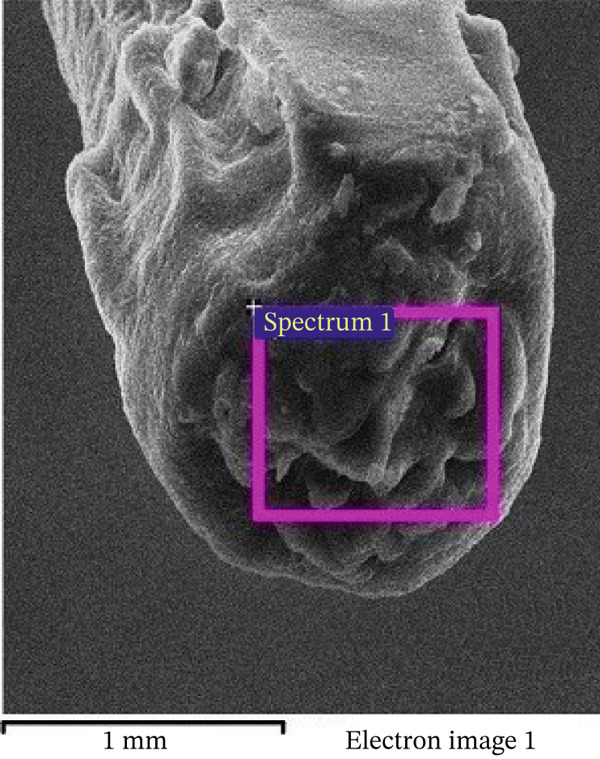
(d)

The fish subjected to microanalysis were collected from the fresh landings at Jetti in the Panaji area in Goa. The worms, after recovery from the fish host, were washed in Hank′s solution and fixed in 4% (w/v) glutaraldehyde buffered with 0.1 M sodium cacodylate (pH 7.4) containing 3% (w/v) sucrose for 4.5 h at 4°C to conduct x‐ray scanning electron microanalysis. The head and body of *R. capoori* and pyloric ceca as well as gill filaments and lamellae of the host fish were processed, but a detectable response emanated only from gill filaments. Therefore, detailed observations were recorded on gill filaments. The roundworms of *R. capoori* collected from the pyloric ceca of *J. dussumieri* were subjected to EDXMA on the appliance model JSM‐6510LV JEOL (Japan) equipped with Oxford Instruments INCAx‐act energy‐dispersive x‐ray analyzer, in conjunction with SEM analysis at USIF (University Sophisticated Instrumentation Facility, Aligarh Muslim University, Aligarh, Uttar Pradesh, India). The analysis of metals as well as the mapping of elements and metals on worms of *A. typica* parasitizing the intestine of *S. sihama* was also done on EDXMA [21]. The specimens were attached to the stubs for preparation of SEM photomicrographs for elaborate analysis of the body parts of the worms and tissues of the host fish. Each body organ of five worms of the same stage of development was subjected to analysis, and these were impinged twice to extract data on elements as well as metals. The results of analysis of metals and elements, namely, Al, As, C, Ca, Cd, Co, Cl, Cr, Cu, Fe, Hg, Mg, Mn, Na, O, Pb, S, Si, and Zn, have been presented in the text and figures along with quantitative data on elements analyzed from the tissues. Various tests of significance, namely, Kruskal–Wallis test [21, 22] and Student′s *t*‐test [23], were calculated, and ANOVA [23] was applied to work levels of significance of the metals accumulated.

### 2.2. Statistical Analysis

#### 2.2.1. Kruskal–Wallis Test

The comparable level of significance was tested using the Kruskal–Wallis test [22]. Three or more independent groups are compared to verify the statistical differences between them. The test works by ranking all the data and comparing the sum of ranks across the different groups to see if they are significantly different [21].

#### 2.2.2. One‐Way ANOVA Analysis

This is the most basic form of ANOVA [23] and is used when there is only one independent variable with more than two levels or groups. It assesses whether there are any statistically significant differences among the means of the groups. It is a parametric statistical method that is used to establish a quantitative difference between the means of three or more groups. This verified the impact of various factors by comparing groups (samples) based on their respective mean.

#### 2.2.3. Preparation of Water Samples for Analysis

The concentration of 3 L of each collected sample of water was determined at 80°C until the volume reached 50 mL. The addition of 4 mL concentrated sulfuric acid (Merck, 98%) was done in each sample, which was digested using the Digesdahl apparatus for 3 min. The addition of 10 mL of hydrogen peroxide (Merck, 30%) was then made, followed by heating to complete oxidation. The samples were filtered separately by a Whatman filter (Merck, 0.45 *μ*m) after cooling. The final volume was made 50 mL with deionized water from the filtrate. The prepared samples were analyzed by the method of EDXMA [24].

## 3. Results

The effort to analyze differentially absorbed heavy metals by various body organs of the newly discovered nematode, *R. capoori*, from *J. dussumieri* demonstrated successfully that the three heavy metals, namely, As, Cr, and Al, exhibited affinity to its extremities on the body, like the exterior surface on the anterior collarette (Figure 1b,d; Table 1). The papillated extremity of the worm also showed affinity to As, as detected in Figure 9 and Table 1. The most remarkable finding of the reported analysis of various body parts of *R. capoori* has been the high concentration of sulfur as a predominant constituent of its inorganic elements, namely, 0.94 wt% of collarette of the worm (Figure 1b; Table 1), 0.76 wt% assessed from within the broken parts of mid‐body of the worm (Figure 7; Table 1), 0.56 wt% in the region of postcaudal cluster of papillae (Table 1), 0.43 wt% reflected from the nonpapillated zone (Figure 11; Table 1) in the vicinity of papillated clusters (Figure 9), and 0.70 wt% in the area of phasmid over the body surface of the roundworm, *R. capoori* (Figures 1c and 6).

**Table 1:** Experimental data on elemental composition analysis of the tissues of body parts of *Johnius dussumieri* infected by *Rotundocollarette capoori*, noninfected fish, and seawater of the Arabian Sea at Goa using energy‐dispersive x‐ray microanalysis (EDXMA) in conjunction with scanning electron microscopy (SEM).

Sl. no.	Element	Head of *Rotundocollarette capoori*	Head of *R. capoori* (en face view)	Posterior part of the body of *R. capoori* with phasmid	Mid‐body of *R. capoori*	Posterior subdorsal papillated part of the body of *R. capoori*	Posterior subdorsal nonpapillated part of the body of *R. capoori*	Part of the skin adjacent to the buccal tooth of *R. capoori*	Buccal tooth of *R. capoori*	Postcaudal collarette of *R. capoori*	Phasmid of *R. capoori*	Seawater of the Arabian Sea at the jetty at Miramar, Goa
		Weight%										
1.	Al	0.16	0.15	—	—	—	—	—	0.42	0.01	—	0.08
2.	As	0.19	—	0.24	—	—	—	—	0.80	—	0.15	0.36
3.	C	64.77	64.17	64.38	63.79	60.90	62.60	28.52	58.81	54.21	65.92	20.52
4.	Ca	—	0.02	—	—	—	—	1.06	—	—	—	0.04
5.	Cd	—	—	—	—	0.11	0.22	—	—	—	—	—
6.	Co	—	—	—	—	—	—	0.21	—	—	2.95	0.02
7.	Cl											22.15
8.	Cr	0.09	0.18	—	—	—	—	1.16	0.24	0.29	1.32	—
9.	Cu	—	—	—	—	—	—	—	1.94	—	0.82	—
10.	Fe	—	—	0.07	—	0.16	—	—	—	—	—	—
11.	Hg	—	—	—	—	—	0.30	—	—	—	—	—
12.	Mg	—	—	0.03	0.09	—	0.34	—	—	0.16	—	9.50
13.	Mn	—	—	—	—	—	—	—	0.24	—	2.46	—
14.	Na	0.36	0.18	—	—	—	0.17	—	—	—	—	6.07
15.	O	33.47	33.55	34.08	34.98	37.95	34.89	—	36.38	44.62	24.28	38.92
16.	Pb	—	—	—	—	—	0.99	4.83	—	—	—	0.70
17.	S	0.73	0.94	0.70	0.76	0.56	0.43	—	0.27	—	—	1.13
18.	Si	0.22	0.55	0.49	0.38	0.32	—	—	—	0.09	—	—
19.	U	—	—	—	—	—	—	3.57	0.91	0.61	2.09	—
20.	Zn	—	0.26	—	—	—	0.07	—	—	—	—	0.50

The pyloric ceca of worms infected by nematodes could drain in 0.94 wt% Pb, but 0.30 wt% Pb was analyzed from the pyloric ceca of noninfected fish. The comparable level of significance as tested by the Kruskal–Wallis test and one‐way ANOVA analysis is given in Table 2. The distal part of the pyloric ceca of worm‐infested fish was found to contain Hg (0.99 wt%), whereas 0.30 wt% was analyzed in the posterior subdorsal nonpapillated part (Figures 11 and 12) of the body of the worms from the same fish, *J. dussumieri*. This provided evidence of the contribution of iron toward additional strengthening to support the functioning of the caudal papillae. The findings got further support from the presence of iron (0.16 wt%) in the papillated region (Figure 9; Table 1) of the same fish as well as in the area around phasmid (Figure 1c; Table 1) in the same fish, *J. dussumieri*.

**Table 2:** Results of one‐way ANOVA analysis to substantiate differential distribution of heavy metals among various organs of *Rotundocollarette capoori*.

Source	Sum of Squares	df	Mean Square	F ratio
*Rotundocollarette capoori* Posterior part Vs mid‐body				
Regression	0.058	1	0.058	19.33*
Residual	0.006	2	0.003	
r = 0.721	*P < 0.05			
*Rotundocollarette capoori* Dorsal Vs Enface view				
Regression	0.087	1	0.087	29.00*
Residual	0.006	2	0.003	
r = 0.841	*P < 0.05			
Body of *Rotundocollarette capoori* Papillated Vs non‐papillated				
Regression	0.041	1	0.041	20.5*
Residual	0.004	2	0.002	
r=0.717 *	P<0.05			

It is remarkable that toxic metals like Cd, Hg, Pb, and As occurred at a greater frequency on and around papillated formations, including the caudal papillae (Figure 9) and phasmid (Figure 5).

### 3.1. Infected Versus Noninfected Fish

The striking difference between the papillated (Figures 9 and 10) and nonpapillated (Figures 11 and 12) postcaudal region of the body of the worm has been the occurrence of iron (0.16 wt%) in the former but not in the latter. The gill tissues of fish in an aquatic body come instantly into contact with the water gushing in. Incidentally, these happen to be the absorbers of the heaviest contaminant metals, like arsenic (0.23 wt%), Cr (to the extent of 0.18 wt% and 0.26 wt%), and Zn in appreciable quantities. The amount of metal absorbed by the gill lamellae was comparable between infected and noninfected fish (up to 0.07 wt%). Arsenic and Zn were absorbed by the tissues of the gills in the nematode‐infected fish but not in the noninfected fish.

In the current investigation, the fact that more than double the content of lead was detected in the liver of infected fish (0.87 wt%) than in the liver of noninfected fish (0.39 wt%) further supports the earlier findings [20]. It has been asserted repeatedly [25] that the greater proportion of strength that the cuticularized exoskeleton derives comes from the greater concentrations of calcium present in the bodily organization of the worm (Figures 1b, c, d, 3, 5, and 7).

The example of the wavering pattern of infectivity during the posttsunami period intriguingly influenced *Paracamallanus* sp. to disappear from *L. malabaricus* in the Arabian Sea after long‐term infestations for several years. Then, all of a sudden, its range of infectivity broadened to encompass a larger variety of fish hosts in the same water body. As follow‐up events, there has been a sharp decline in *L. malabaricus* populations. Simultaneously, *Paracamallanus* infections transitioned from this fish, demonstrating host‐switching, and commonly occurred in another cohabitant, *J. dussumieri*, in which these nematode infections were previously uncommon. A diversity of faunal species, leading to the emergence of *R. capoori*, also co‐occurred. Simultaneously, highly advanced anisakid larvae, particularly of *Contracaecum* sp., also occupied a newer niche, within *L. malabaricus*. These add‐ons could well bring to notice the environmental influence to produce alterations in the pattern of infectivity of bioindicator parasites. However, the resultant enhanced host range of these helminth parasites warrants the use of the term “euryparasitic” for such nematodes; conversely, conventionally distributed roundworms, like *Paracamallanus* sp. and *Iheringascaris goai* [26] reported thus far, exhibit a susceptibility level within a restricted range of hosts. These could be termed as “stenoparasitic” forms [1, 26, 27]. The finding of *Neolebouria* [28] (Opecoelidae: Plagioporinae) from the whitecheek monocle bream, *Scolopsis vosmeri* (Perciformes: Nemipteridae), inhabiting the Panjim coast at Goa, established its retention in perciform fish in the Arabian Sea, where a warmer thermal regime (up to 32°C) prevailed. Simultaneously, the delineation of this stock from as many as 22 species of the genus *Neolebouria* that parasitize deep‐sea fish of the Atlantic and Pacific Oceans in Europe and America was published [29].

The potential of helminths for the effective absorption of bile‐associated lead transported within the intestinal vessels of the fish hosts resulted in a fruitful operational resistance, preventing the absorption of lead into the intestinal tissues of the host. This was experimentally illustrated [20] in chub, *Leuciscus cephalus*, under infections by the acanthocephalan, *Pomphorhynchus laevis*. Thus, the cycle of reabsorption of lead within the wall of the intestine is interrupted through alterations in the biochemical and physiological elements of the hepatic portal transport system.

### 3.2. Influence of Hydrobiological Factors

The results on correlations of water chemistry with infections by helminths in marine fish from the Arabian Sea (the area of current investigations) published by the authors and coworkers earlier [10] emphasized that a high content of hardness (11,000 mg/L) affected the extent of helminth infestation as well as their prevalence.

#### 3.2.1. Environmental Sink

The similarity to a sink is preferred in this environmental context because of the nematode′s potential to sequester toxic metals, thereby protecting the host fish′s tissues from the detrimental effects of metal toxicity. Since the endoparasitic helminth survived inside the intestinal environment of the host fish even after absorbing the toxic chemicals, which could otherwise have harmed the host fish, it acted as a savior of fish from metal toxicity in the ambient aquatic environment of the fish host.

The sulfur ion contributors are the disulfide bonds in the twin amino acids, cysteine and cystine. In infected pyloric ceca, 0.14 wt% Pb was detected, while the Pb concentration in water was 0.70 wt%; notably, 0.99 wt% Pb was encountered in *R. capoori* (Figure 17). Noticeably, high sulfur content was a common occurrence in various components of *R. capoori* as well as in the water body (Table 1). These specifically contributed to the sturdy feature of the head armature which is the specific characteristic of the worms.

The finding of 0.99 wt% Pb in *R. capoori* evidently indicated that Pb from water (Figures 16 and 17) was drained by fish at the site of infection, that is, pyloric ceca. The parasite thereafter accumulated maximum Pb from pyloric ceca (0.99 wt%) (Table 1) that has been reported in this study.

Mapping via the EDXMA technique provided a quantitative assessment of the Pb and Fe content at a depth of 2 mm inside the buccal tooth element of *A. typica* (Figure 18). The content of elements carbon and oxygen was also mapped, as presented in Figure 19. The segregated content mapping of the distribution of Pb (Figure 20) and Fe (Figure 21) was also done.

## 4. Discussion

### 4.1. Infected Versus Noninfected Fish

There was no quantitative detection of Zn available in the infected distal part of the pyloric ceca, but the amount of Zn was 0.06 wt% in the infected proximal part of the latter. However, conversely, 0.30 wt% Zn was detected in the noninfected distal part of the pyloric ceca. These values corroborated the substantially lower values (0.06 wt%) of Zn in the proximal part of pyloric ceca, which was the site of infection of *R. capoori*, but was simultaneously absent in the distal part of pyloric ceca as published earlier [24]. The latter was the evidence of absorption of Zn from the tissues of the distal part of the ceca that was infected and hence occurred in the tissues of the nematode, *R. capoori*.

The occurrence of iron in the papillated than nonpapillated postcaudal region of the body signifies the utility of iron and its functional importance to undertake the assistance in reproductive processes, as required by the worm, because the papillae in the caudal region are known to perform the function, particularly to assist in the copulatory process. The specialized cordons of acuariids were specifically attributed to their inordinate strength due to the presence of S, Ca, and P within the nematodes [25]. In recent years, the functioning of naturally occurring sulfur‐bound ligands has been proposed to trigger protectivity [30] under natural environments.

The conclusions [24] were upheld, reasserting that the differences in metal dissolution within the tissues of the gills (*Y* = 2.882 + 1.172*X*, *p* < 0.001; *F*_1,2_ = 21.67; *r* = 0.581; *p* < 0.015) and the pyloric ceca (*Y* = 4.808 + 0.716*X*; *F*_1,4_ = 12.932; *r* = 0.705; *p* < 0.001) of infected vis‐à‐vis noninfected fish were significant, as determined by one‐way ANOVA analysis and Kruskal–Wallis test (Table 2):

Gill filament: Probability is 0.50, assuming chi‐square distribution with 5 df.

Pyloric ceca: Probability is 0.66, assuming chi‐square distribution with 6 df.

The majority of elements in worm tissues and water samples from the sites these were beyond the acceptable limits of drinking water standards proposed in [31–34].

**Figure 2:**
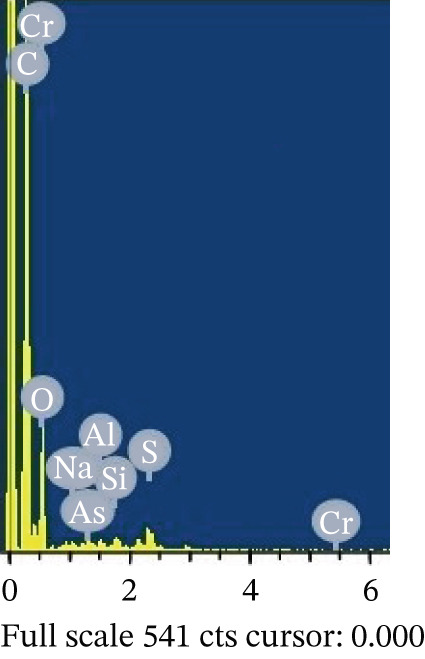
Head of *Rotundocollarette capoori* (dorsal view) for observed chemical elements after a 50‐s run including specific peaks of different elements.

**Figure 3:**
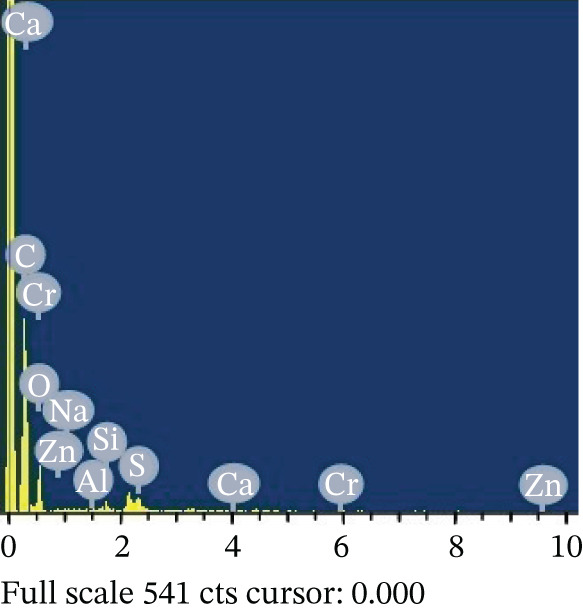
SEM illustration of Figure 1a for observed chemical elements after a 50‐s run including specific peaks of different elements.

**Figure 4:**
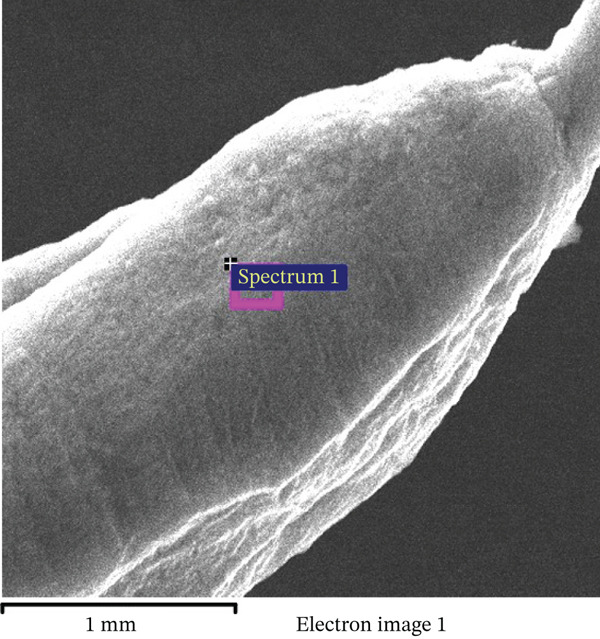
SEM illustration of Figure 1b for observed chemical elements after a 50‐s run including specific peaks of different elements.

**Figure 5:**
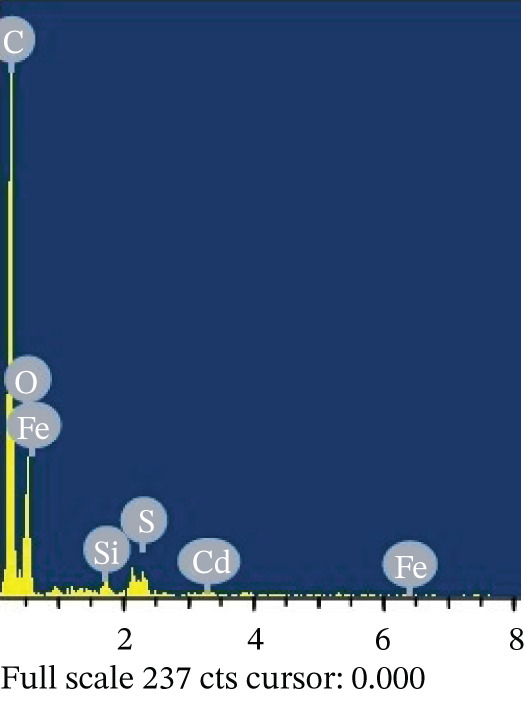
Head of *Rotundocollarette capoori* (Phasmid) for observed chemical elements after a 50‐Sec run include specific peaks of different elements.

**Figure 6:**
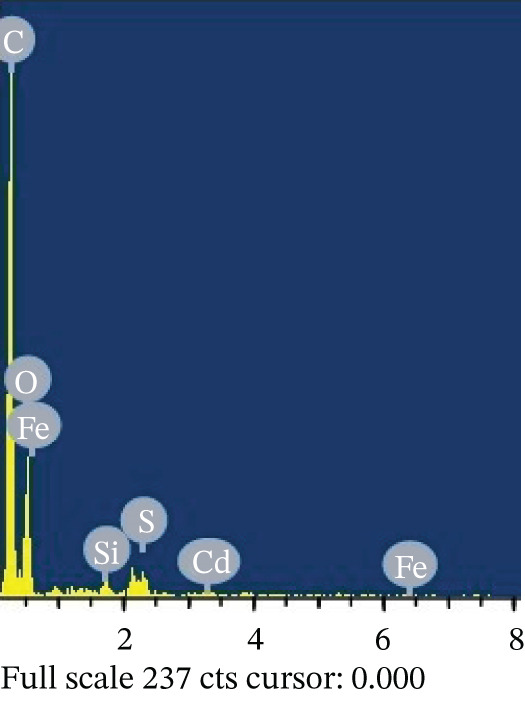
Head of *Rotundocollarette capoori* (Enface view) for observed chemical elements after a 50‐Sec run include specific peaks of different elements.

**Figure 7:**
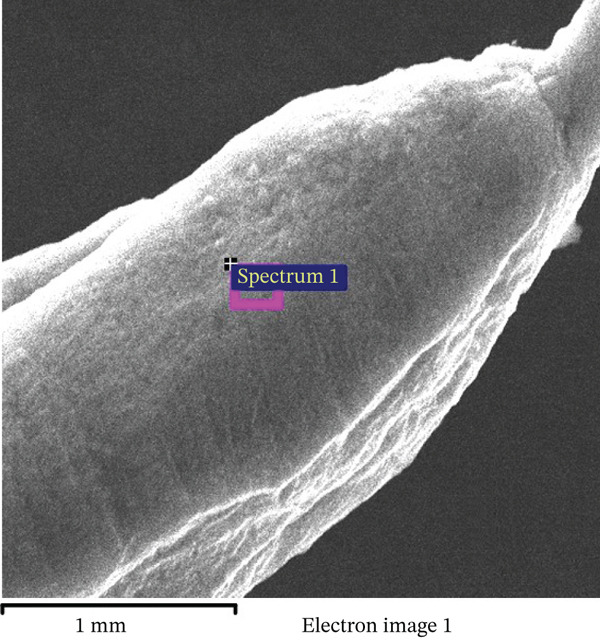
Mid‐body of *Rotundocollarette capoori* for observed chemical elements after a 50‐Sec run include specific peaks of different elements

**Figure 8:**
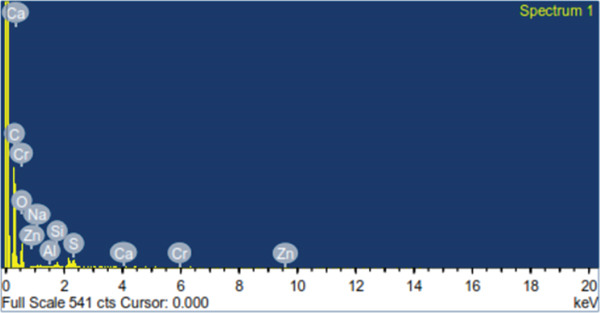
Skin of mid‐body of *Rotundocollarette capoori* for observed chemical elements after a 50‐Sec run include specific peaks of different elements.

**Figure 9:**
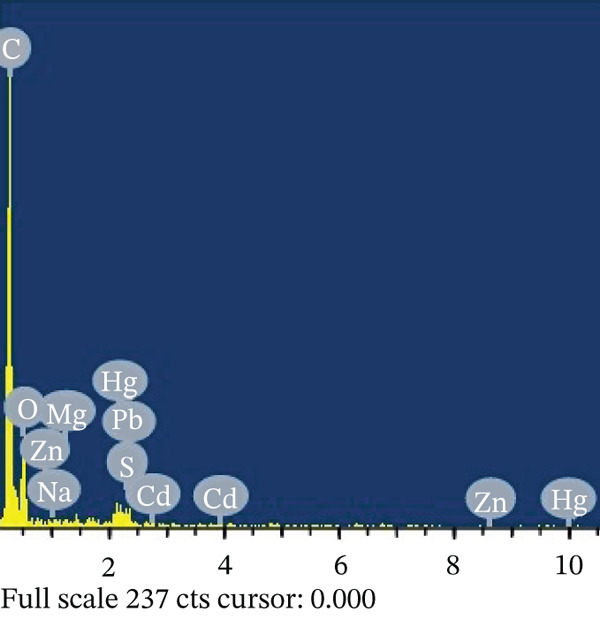
SEM of the posterior subdorsal papillated part of the body of *Rotundocollarette capoori* (the EDXMA‐generated purple square represents the measured area).

**Figure 10:**
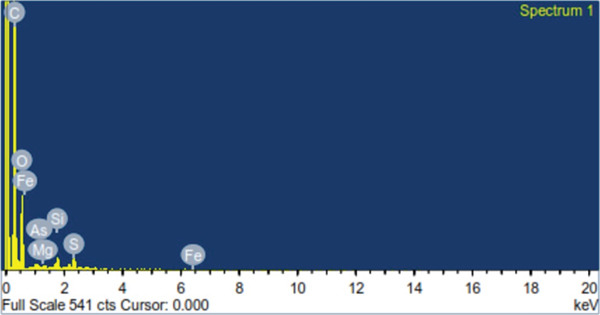
A posterior subdorsal papillated part of the body of *Rotundocollarette capoori* for observed chemical elements after a 50‐s run including specific peaks of different elements.

**Figure 11:**
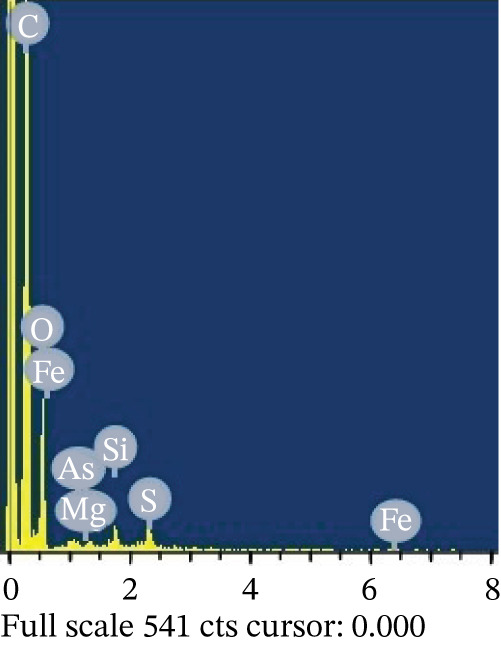
SEM of the posterior subdorsal nonpapillated part of the body of *Rotundocollarette capoori* (the EDXMA‐generated purple square represents the measured area).

**Figure 12:**
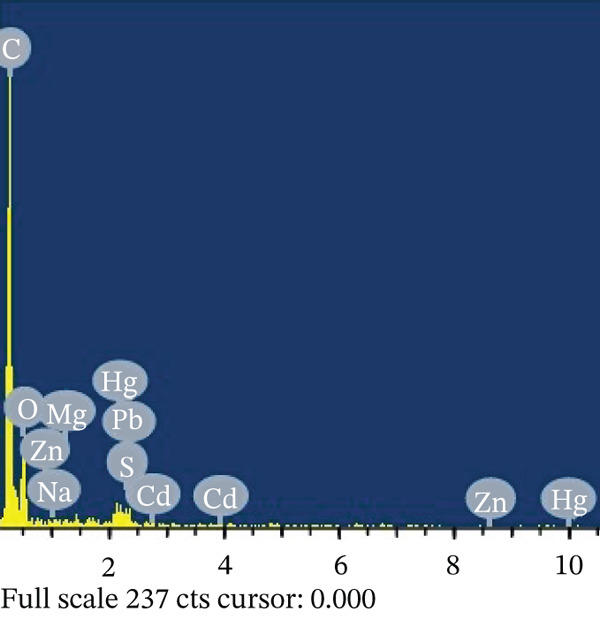
A posterior subdorsal nonpapillated part of *Rotundocollarette capoori* for observed chemical elements after a 50‐s run including specific peaks of different elements.

**Figure 13:**
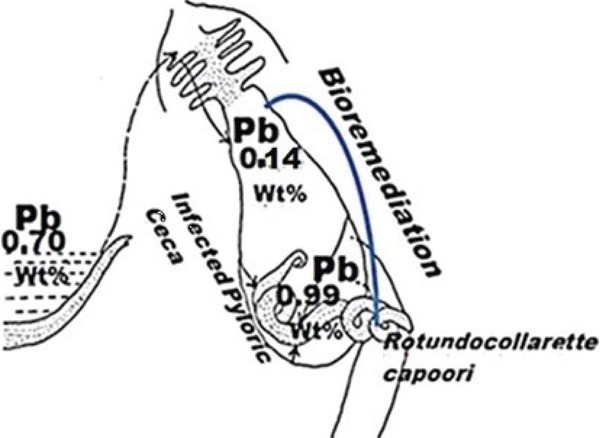
Microphotograph of SEM of the water sample from jetty at Site 1 in the Arabian Sea.

**Figure 14:**
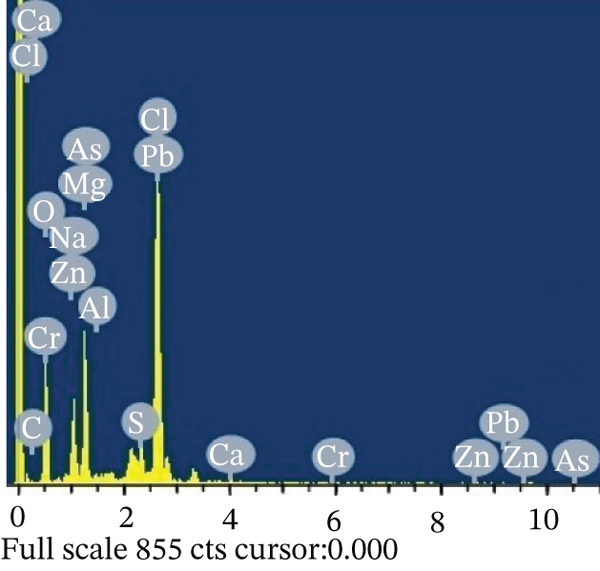
Microphotograph of SEM of the water sample from jetty at Site 2 in the Arabian Sea.

**Figure 15:**
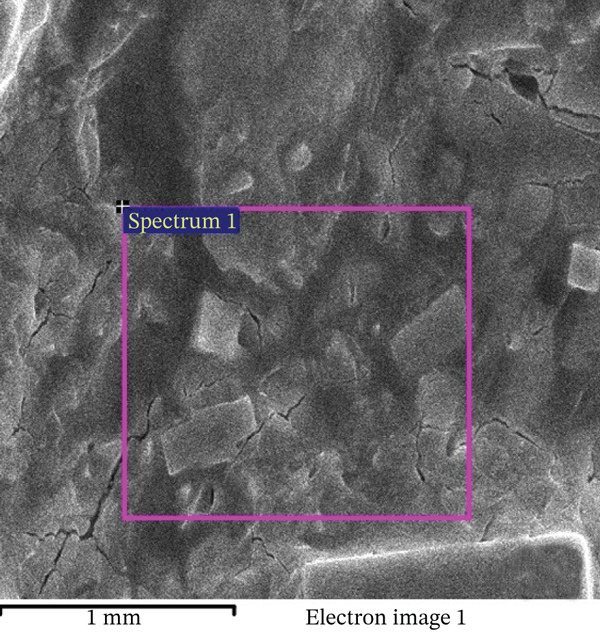
Analysis of the water sample from jetty at Site 1 in the Arabian Sea to observe chemical elements after a 50‐s run including specific peaks of different elements (the EDXMA‐generated purple square represents the measured area).

**Figure 16:**
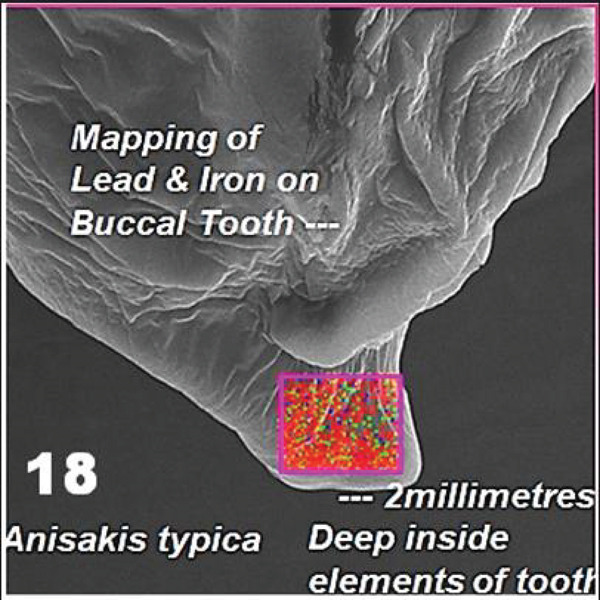
Analysis of the water sample from jetty at Site 2 in the Arabian Sea to observe chemical elements after a 50‐s run including specific peaks of different elements (the EDXMA‐generated purple square represents the measured area).

**Figure 17:**
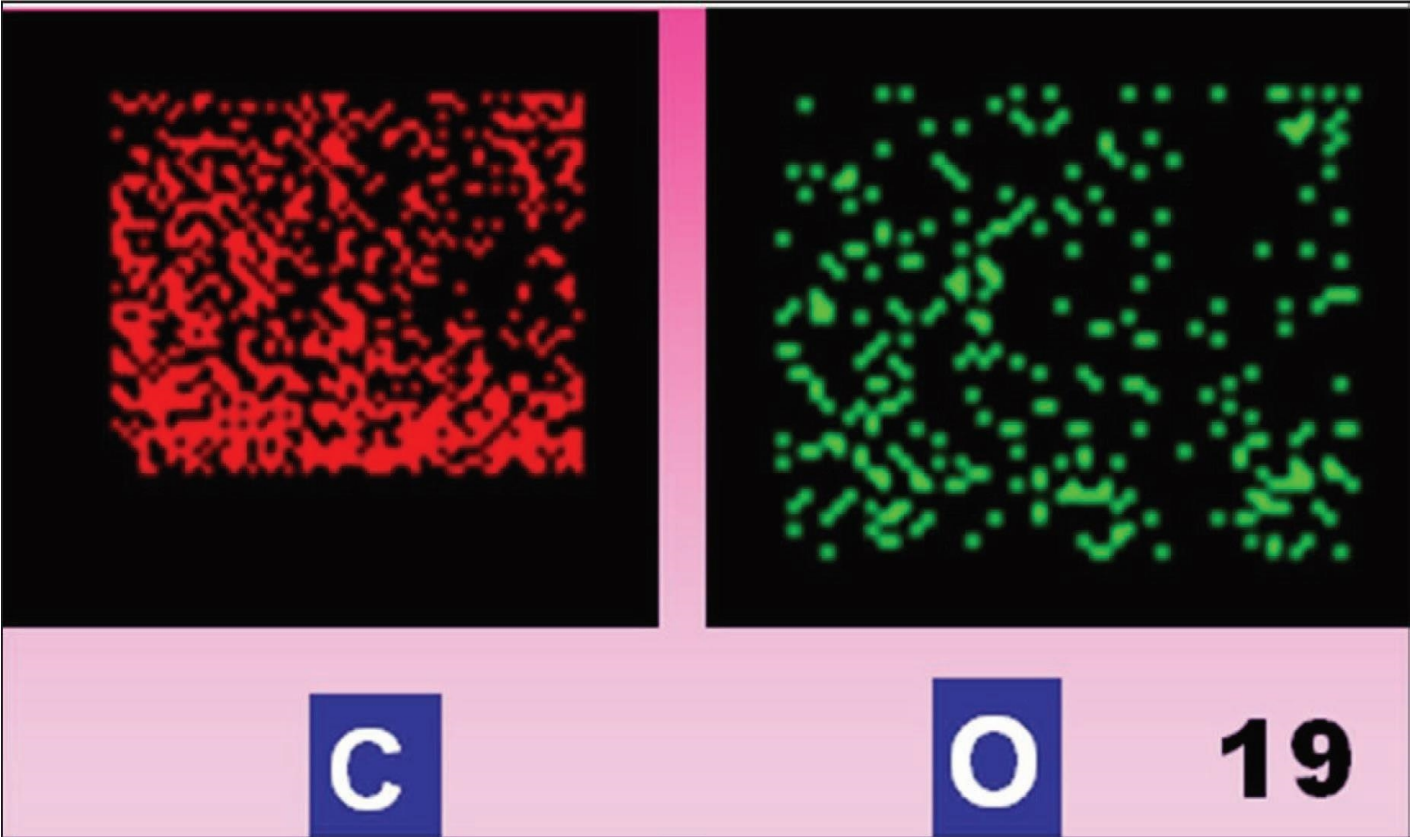
Diagrammatic representation of the transfer pathway of biometallic movement for bioremediation through Rotundocollarette capoori in an aquatic ecosystem.

**Figure 18:**
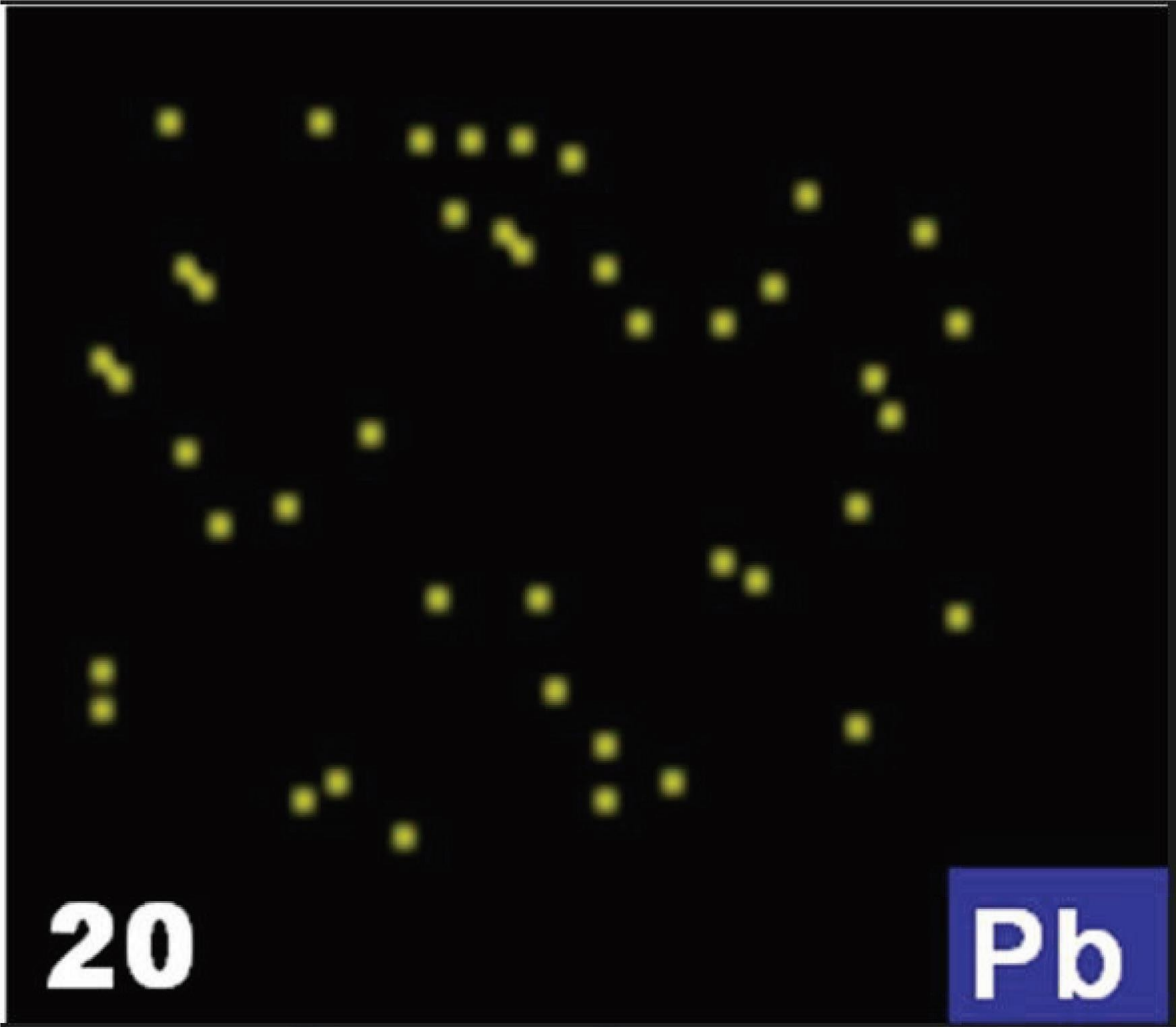
Mapping of biometals on the dorsal tooth of *Anisakis typica* parasitizing the reef‐associated fish, *Sillago sihama*.

**Figure 19:**
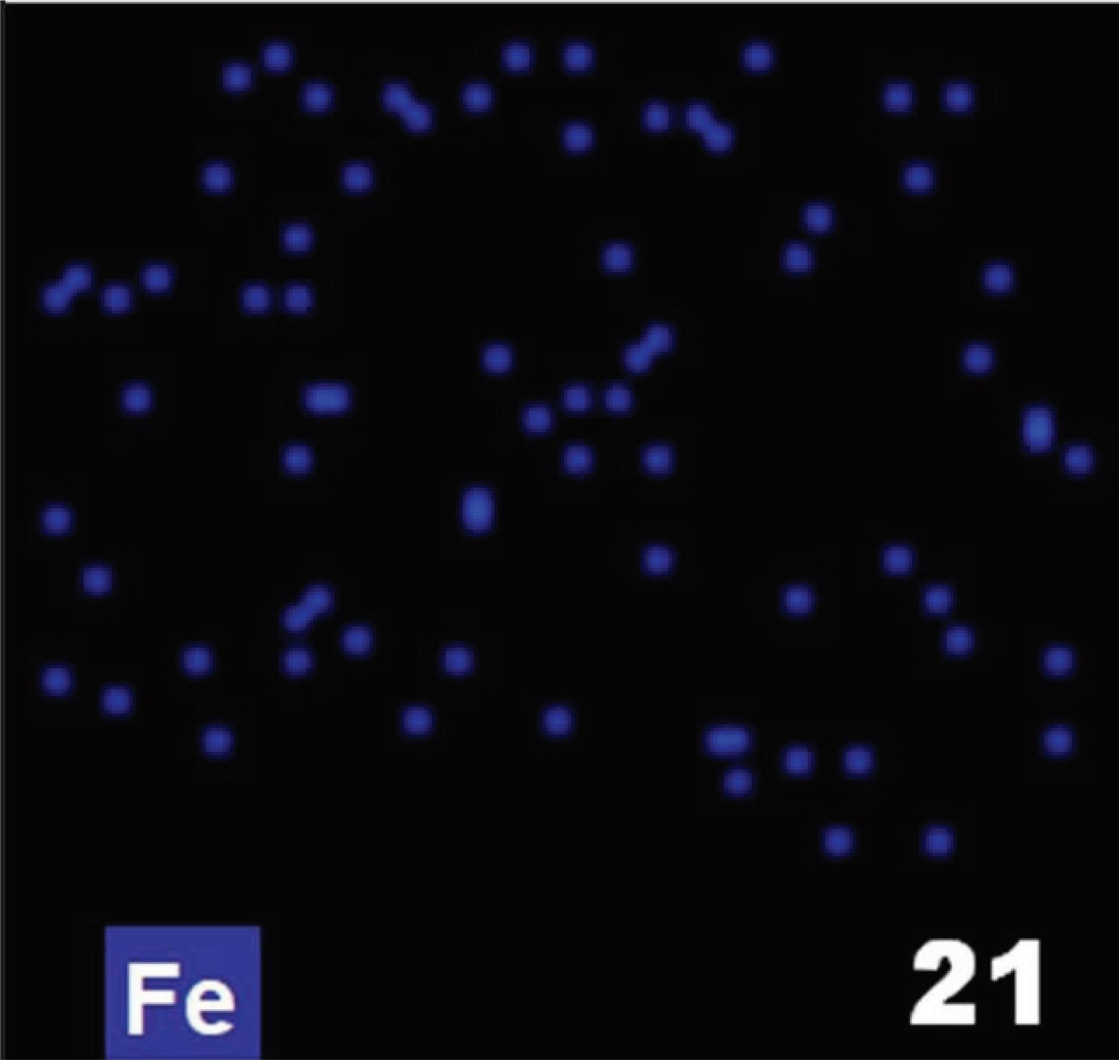
Mapping of elements (magnified) on the dorsal tooth of *Anisakis typica* parasitizing the reef‐associated fish, *Sillago sihama*.

**Figure 20:**
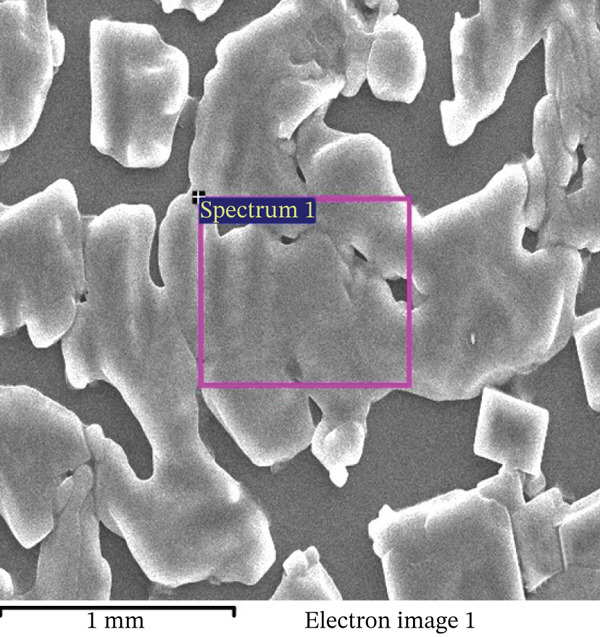
Mapping of lead (magnified) on the dorsal tooth of *Anisakis typica* parasitizing the reef‐associated fish, *Sillago sihama*.

**Figure 21:**
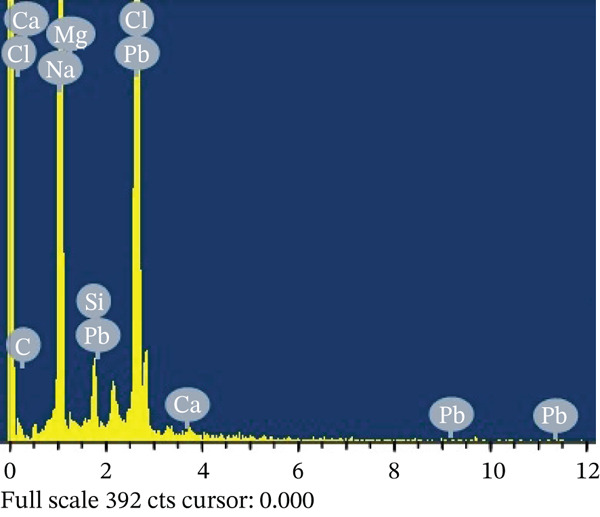
Mapping of Iron (magnified) on dorsal tooth of *Anisakis typica* parasitizing reef‐associated fish, *Sillago sihama*.

### 4.2. Influence of Hydrobiological Factors

In the context of the present investigation, the influence of metal toxicity declined under augmented hardness of the water [35]. The content of manganese was highest (2.46 wt%) on the surface of phasmids in the postcaudal region of the worm (Table 1). Though this did not exceed the limits beyond which damage could be caused to the molecular mechanisms in the body of nematode, mitochondrial dynamics, autophagy, and antioxidant defense systems could be under stress due to toxicity induced by manganese [36,37] attributed strength and noticeable hardness to the tissues of phasmid for its support in the copulatory role in the postcaudal region where excess of cobalt (2.95 wt%) was encountered.

Literature recorded the declining influence of increased hardness of seawater on the adverse effects of copper [38–40], cadmium [41], and zinc [40]. The metallic ion constituents compete with Ca^++^ and Mg^++^ ions in water bodies with hard water and search to occupy uptake sites on the external surface of parasitic organisms. Identical conclusions were drawn by the investigations [35] on Egyptian fish to settle the role of parasites of fish as metal sinks which apparently assisted in improving the health of fish in farmed fish.

It is concluded that the nematode community provides bioremediation by accumulating heavy metals like Hg, Zn, Pb, and iron within their soft body tissues inside the fish host. Thus, the fish survived despite the heavy metal pollution. Their probable association with acanthocephalan egg shells and hooks was emphasized [42]. The natural bioremediator characteristics displayed by the zoonotic anisakid *R. capoori* closely resembled those of *Ascaris* sp. and *Echinocephalus* sp. in the study [43] that highlighted these parasites as sensitive indicators of the presence of metal contaminants, like Pb, Cd, Hg, As, Zn, and Fe.

The findings on the strengthening of the caudal papillae were further supported by the presence of iron (0.16 wt%) in the papillated region (Figure 9; Table 1) of the same fish host, *J. dussumieri*, as well as in the area around phasmids (Figure 1c; Table 1).

The findings [44] determining a higher yield of sulfur illustrated the supportive piercing action of parasitic extremities to push through within the tissues of the alimentary canal. On similar terms, the highest quantitative presence of sulfur (0.94 wt%) in the head region of the nematode, *R. capoori*, corroborated the strength and rigidity assigned to the head region to make way beyond stiffer muscular and other tissues within the cavity of the pyloric ceca.

The positive influence on the health of the fish hosts, achieved by utilizing the heavy metal filtering capacity of the iron absorbed by the parasites, was asserted [45]. Recently [46], investigations have also demonstrated the affinity of iron for the body tissues of alligators in the aquatic environment. The closest finding to the present study was the one [47] that was conducted on third‐stage larvae of *Contracaecum*, a member of Anisakidae. These larvae accumulated metallic iron with properties to be transformed into two organic forms, similar to the Fe reported from the camallanid worms, *Procamallanus* [48].

The bile acid release in the vicinity of the harbored site (pyloric ceca) of parasitic invasion [20] was attributed as a valid reason for the occurrence of higher iron content in this area, occupied by *Hysterothylacium reliquens* within the alimentary canal of fish [20]. The altered physiology of the vertebrate host due to the excessive iron influx within the intestine provided an opportunity for the nematodes to absorb additional iron content to which the host tissues were exposed at the onset of helminthic invasion. The comparable depletion of iron in the infected intestine was the obvious sign of the metal iron filter from the infected intestine to the worm [20].

Further evidence of the rapid accessibility of iron to the heme group than to FeSO_4_ [49] was provided [50] at the onset. The whole process, therefore, contributed to the restoration of the drained iron pools of serum ferritin of the parasitized fish host. The critical inputs [16] illustrated that the iron content available in the surrounding medium of fish, in the aquatic body, like a pond, could first be drained into the body organs of the host. Now, from these organs that were infected, the roundworms drained the absorbed iron content into their own body through the metallic filter, as narrated above [45]. These findings might revolutionarily be indicative, primarily of remitting indicator response by the nematodes. Next, the influx of environmentally available iron in the fish microenvironment through the metallic filter could trigger an amending effect on the former response as the nematodes exploited the moderately available concentrations of iron and other heavy metals in the fish microenvironment for their development, growth, and survival [16].

The assertions, therefore, emphatically elaborated that the contention has gained ground for helminth parasites as being accumulator effectors under the category of bioindicators in the Indian aquatic environments [47]. Long‐term investigations [1, 51] could make secure assumptions to conclude on the pattern of infectivity even when nematode populations are confronted with major upheavals, like a tsunami. A valid reason for occurrence for the higher iron content in this area was attributed to the parasite in an earlier report [20], because infectivity by *H. reliquens* within the alimentary canal of fish contributed to higher iron content [48].

#### 4.2.1. Environmental Sink

Lead from water in the vicinity of the fish was transported to the pyloric ceca, from where the worms sequestered the maximum concentration of Pb from the pyloric ceca into the parasites. Apparently, Pb from the water at the Jetti (Figure 13: Pb, 0.75 wt%; Figure 15: Pb, 0.70 wt%) was drained into the system of fish (noninfected pyloric ceca: Pb, 0.30 wt%).

The tissues of the proximal part of the pyloric ceca in the infected fish absorbed an exceptionally higher amount of metal, that is, Hg (0.94 wt%), under the accumulator effect. But, since Hg was not detected from the marine water in the ambience, apparently, the metal was drained by the parasitic roundworm from the organ tissues of the fish host through which the nematode wandered and finally migrated into its final site of infection, that is, the pyloric ceca. The metal content 0.30 wt% of Hg detected (Table 1) from the posterior subdorsal nonpapillated part of the body of *R. capoori* could apparently be explained on account of Hg having been drained in from the distal part of the pyloric ceca of *J. dussumieri* that was infected by *R. capoori* and where Hg was detected in appreciably higher quantity (0.94 wt%).

The likely correlation of sulfur with the stiffness of head armature matched the skeletal strength of mammalian hosts, placed higher in the ladder of evolution, while these worms parasitized host fishes of the lowest order in the evolutionary series. The probable association between acanthocephalan egg shells and hooks was emphasized in recent years [42]. Certain heavy metals acted as a metal filter to block the ill effects of accumulated metals within the parasite [52]. Thus, the improvement in fish health was also brought to record. It was also asserted [53] that the biometals were absorbed by the parasites harbored within the fish host and this saved the host from the ill effects of toxic metals.

A recent report on the spinated infrastructure on the body of *Aplectana* [54] vis‐à‐vis its comparison with EDXMA of the muscular papillated growth on the external skin of *R. capoori* peculiarly recorded the presence of sulfur as the chemical constituent of both papillated and spinated projections both on *R. capoori* and *Aplectana*.

The emergence of diagnostic value characteristics in the worms of *Aplectana* has been investigated [54] recently by the authors and coworkers. This brought to the limelight the specialized spinated infrastructure on the body of *Aplectana* and its comparison with the outcome of EDXMA of muscular papillated growth on the external skin of *R. capoori*. The peculiarly recorded presence of sulfur as the common chemical constituent of both papillated and spinated projections on *R. capoori* as well as *Aplectana* specifically contributed to the detection of their role as bioindicators [55].

One of the favorable components of coastal and estuarine fisheries in India is Indian sand whiting, *S. sihama* (family: Percomorphoidea). The helminth fauna of this fish in the Indian Ocean has been scarcely investigated [26]. However, commercial utilization of the marketed fish in the form of dried, smoked, or frozen fish is in common practice [56]. The popular Japanese sushi outlets and frequent consumption of fish meal at cheap kaitenzushi (conveyor belt sushi restaurants) have ensured effective encroachment into the feeding habits of Indians. The production of fish meal using Indian mackerel (*Rastrelliger* spp.) facilitated the mechanism of transmission of nemic larval and developmental stages around coastal areas of the Bay of Bengal in the Indian Ocean, the southern coast of East Java, and Bangladesh [57, 58]. The Kerala diet prominently comprises both fresh and dried fish because Kerala, being a coastal state, was the leading state as well as a leading fish producer of the country (Tables 1, 2 and 3) [59]. A total per capita fish consumption, 2.26 kg (rural) and 2.21 kg (urban), is on record in Kerala state [60].

**Table 3:** Distribution of bioaccumulation factor (BAF) in the extremities of the body of the anisakid worm *Rotundocollarette* infesting *Johnius dussumieri* at Ilha Grande Island, Goa.

Sl. no.	Metal/element	Head of *R. capoori* (en face view)	Posterior part of the body of *R. capoori* with phasmid	Mid‐body of *R. capoori*	Part of the skin adjacent to the buccal tooth of *R. capoori*	Buccal tooth of *R. capoori*	Postcaudal collarette of *R. capoori*	Phasmid of *R. capoori*
		Weight%						
1.	Al	1.875	—	—	—	5.25	1.25	—
2.	As	0.528	0.667	—	—	2.22	—	0.417
3.	Ca	0.5	—	—	26.5	—	—	—
4.	Co	—	—	—	10.5	—	—	128.26
5.	Cr	—	—	—	1.16	—	—	—
6.	Mg	—	0.003	0.009	—	—	0.017	—
7.	Pb	—	—	—	6.9	—	—	—
8.	S	0.832	0.619	0.673	—	0.239	—	—
9.	Si	0.236	0.210	0.38	—	—	0.039	—

### 4.3. Limitation

The stringent, restricted absorbance of biometals not exceeding a depth of 2 mm in body tissues was a technical limitation of the instrumentation beyond the control of the investigating team.

## 5. Conclusions

The greater ion accumulation of calcium, sulfur, and phosphate in the roundworm tissues of *R. capoori* projected an accumulator effector indicator phenomenon, which upholds the strengthening influence of cuticularized components within the nemic body organization. The lowered contaminant metal concentration in the specific organ at the site‐specific habitation of the roundworms, that is, the proximal part of the pyloric ceca, strengthened the possibility of transfer of biometallic toxins from the host′s body tissues to parasitic body tissues, so that the detoxified ambience outside the microenvironment of the worms ensured a contaminant‐free metabolic environment for the fish tissues to develop in a healthy state. This study noted the unique strength of absorption of contaminants and toxicants by the invasive species groups of raphidascaridid nematodes, *R. spinicaudatum*, that exhibited an intermixing of riverine populations with invasive marine nemic populations from the Arabian Sea.

Contaminant bioaccumulation from fish hosts by parasitic nematodes resulted in augmented fish fitness. These intestinal fish parasites proved to be benefactors to their fish hosts, counteracting the effects of metallic constituents and trace elements in the ambient environment by adopting the role of bioindicators. These may resolve the intricacies of environmental interactions and parasitic ecosegregation.

## Author Contributions

Conceptualization: Sandeep K. Malhotra; collection and investigation: Anshika Yadav and Anita Yadav; writing—original draft preparation: Sandeep K. Malhotra; writing—review and editing: Payal Mahobiya and Anita Yadav; visualization: Anshika Yadav and Anita Yadav.

## Funding

No funding was received for this manuscript.

## Disclosure

All authors read and approved the final version of the manuscript.

## Conflicts of Interest

The authors declare no conflicts of interest.

## Data Availability

The data that support the findings of this study are openly available in Zoological Survey of India at https://zsi.gov.in (Reference Number A/23105).
